# A triple-blind randomized clinical trial of different associations between dexamethasone and non-steroids anti-inflammatories for preemptive action in third molar extractions

**DOI:** 10.1038/s41598-021-04068-z

**Published:** 2021-12-27

**Authors:** Gustavo Antonio Correa Momesso, Gustavo Augusto Grossi-Oliveira, William Phillip Pereira Silva, Renan Akira, Fernando Chiba, Tárik Ocon Braga Polo, Tiburtino José de Lima Neto, Bárbara Ribeiro Rios, Ana Paula Farnezi Bassi, Doris Hissako Sumida, Michael Han, Michael Miloro, Leonardo Perez Faverani

**Affiliations:** 1 Department of Diagnosis and Surgery, School of Dentistry, São Paulo State University (UNESP), Rua Jose Bonifacio, 1193, Araçatuba, SP 16015-050 Brazil; 2 Department of Basic Sciences, School of Dentistry, São Paulo State University (UNESP), Araçatuba, SP Brazil; 3 Department of Oral and Maxillofacial Surgery, UIH Medical Center; College of Dentistry, University of Illinois at Chicago (UIC), Chicago, IL USA

**Keywords:** Clinical trial design, Oral analgesics

## Abstract

The aim of this study is to evaluate the preemptive analgesic effects of dexamethasone (DEX) alone or combined with non-steroidal anti-inflammatory drugs (NSAIDs) in third molar surgeries. The subjects were divided into five groups (n = 20 teeth/group); subjects received only 8 mg of dexamethasone 1 h before the surgical procedure (DEX group), or in combination with etodolac (DEX + ETO), ketorolac (DEX + KET), ibuprofen (DEX + IBU), loxoprofen (DEX + LOX). Paracetamol 750 mg was provided as the number of rescue analgesics (NRA). Salivary PGE2 expression was measured preoperatively and at 48 h. Edema and Maximum mouth opening (MMO) were measured postoperatively at 48 h and 7 days. A visual analog scale (VAS) was performed postoperatively at 6, 12, 24, 48, 72 h, and 7 days. Salivary expression of PGE2 showed a decrease only for the DEX group. Edema and MMO and NRA consumption showed no significant differences among the groups (*P* > 0.05). The VAS showed a significantly lower pain perception at 6 h after the surgery for the DEX + ETO and DEX + KET groups (*P* < 0.05). The combination of DEX and NSAIDS should be considered for preemptive acute postsurgical pain management in third molar surgery. In some drug associations such as dexamethasone 8 mg + NSAIDS (ETO and KET) in the pre-operative time, only a few rescue analgesics are necessary.

## Introduction

Postoperative pain and edema remain significant concerns for patients who undergo third molar removal. For this reason, the literature contains several studies about controlling these symptoms^1^. It is the responsibility of the surgeon to treat the postoperative symptoms of challenging third molar surgeries, which can be quite intense, limiting patients’ daily routines, and causing considerable discomfort^2,3^.

Thus, preoperative drug therapies have been suggested to prevent or relieve postoperative symptoms^3,4^. Preemptive analgesia consists of managing drugs prior to nociceptive stimulation and is defined as an anti-nociceptive treatment that prevents the inflammatory response from stimulation of the afferent pathway to the central nervous system. This general mechanism is responsible for the amplification of the postoperative pain response^5^.

Steroidal anti-inflammatory drugs (e.g. corticosteroids) are used extensively during the preoperative period prior to third molar surgery. Several authors have demonstrated the efficacy of these drugs, mainly in the control of postoperative edema and trismus (MMO)^6–9^. This effect is achieved because corticosteroids inhibit the conversion of phospholipids into arachidonic acid by phospholipase A2. This mechanism is responsible for preventing the production of leukotrienes, prostacyclins, prostaglandins, and thromboxane A2, which mediate pain and inflammation. The use of corticosteroids is believed to inhibit the initial step in this process^6^.

Nonsteroidal anti-inflammatory drugs (NSAIDs) are mostly used during the postoperative period of third molar surgeries due to their considerable anti-nociceptive effects^10–12^. Its mechanism of action consists of inhibiting the cox enzymes, in a specific or non-specific way depending on the drug of this selected class, leading to a decrease in the production of prostaglandins, in addition to inhibiting specific proteinases involved in the degradation of proteoglycans and cartilage collagen, they also inhibit the production of oxygen radicals and bradykinin release, as well as the lymphocyte response to antigenic stimulation, phagocytosis, and granulocyte and monocyte chemotaxis consider inflammation and its symptoms considerably^13^. However, recent studies have demonstrated that the preemptive combination of NSAIDs and corticosteroids seems to improve the relief of postoperative symptoms after third molar surgeries, mainly in the acute postoperative period^4,14,15^. A possible explanation for the efficacy of this combination is that when administered before surgical trauma and the onset of pain, the drugs will be present in the bloodstream at the appropriate levels, and at the appropriate time when pain symptoms begin. In addition, steroidal anti-inflammatory drugs act at the beginning of the inflammation cascade, preventing the formation of arachidonic acid, while non-steroidal anti-inflammatory drugs act by preventing the conversion of arachidonic acid, which by chance was synthesized, into prostaglandins, preventing a succession of cascade events. Thus, it seems appropriate to assess which preventive therapies are most effective and whether their combined use offers any additional benefit by acting at two different times in the inflammation cascade^16^.

Since there is a lack of consensus and few studies regarding the preemptive combination of steroids and NSAIDs aimed at improving the postoperative pain symptoms after third molar surgery, the purpose of this study is to compare the preemptive use of dexamethasone alone, and the combination of dexamethasone with different NSAIDs, in third molar surgeries.

## Methods

### Ethical considerations

This prospective, randomized, triple-blind clinical trial was approved by the Ethical Committee for Human Experimentation from the São Paulo State University—School of Dentistry, Araçatuba—São Paulo, Brazil (number # 88,903,518.4.0000.5420) in 2018. The trial was registered in a Brazilian Clinical Trials Registry (REBEC—date of registration: 14/02/2019—number of registration: #RBR-8rfwnq) and was written in accordance with the CONSORT guidelines (www.consort-statement.org) and the principles for medical research involving human subjects in the 1963 Declaration of Helsinki^17^. All subjects received the research information through the informed consent process. The patients were initially informed that the research aimed to compare many different drugs usually used in relieving the inflammatory symptoms and pain, and they were not aware about what the drugs were prescribed for. Besides that, all the risks about the surgical procedure were informed such as local and systemic infections, alveolar inferior nerve and lingual paresthesia, facial nerve paralysis due to anesthetic technique, mandibular fracture, bleeding, and other minor risks. After this, the patients were instructed that they could quit the research at any moment. These terms explained the study objectives and justifications, the benefits and risks to which the subjects were exposed, and the other items described in the National Health Council’s Guidelines (Resolution CNS 466/12).

### Patients and subjects

This study involved 64 subjects presented as healthy subjects fit with the inclusion criteria (ASA I-II: According to those classified by the American Society of Anesthesiologists ASA II are patients with mild systemic disease. Example: Patient with no functional limitations and a well-controlled disease (eg, treated hypertension, obesity with a BMI below 35, frequent social drinker or cigarette smoker). For this, the authors performed an adequate pre-operative anamnesis, as well as requesting laboratory tests and even medical evaluations, when necessary, to define whether the patient had no functional limitations due to mild comorbidity.), never-smokers, between the ages of 16 and 35 years, with an indication of removal of the mandibular third molars with at least 2/3 of the root formed (Class I or II; Position A/B from Pell and Gregory). All surgical procedures required bony removal and tooth sectioning in order to extract the impacted third molars.

Patients evaluated might present with the inclusion criteria established by the authors, which were:

- Healthy patients with no local and systemic disorders.
- Aged between 16 and 35 years.
- Need of removal lower third molars with at least 2/3 of root formation, evaluated by radiographic exam.

Besides that, patients which did not present the necessary characteristics to participate in the study were excluded. The criteria were:

- Patients presented lower third molars on C position, according to Pell & Gregory classification.
- Local clinical signs which could not indicate the surgical procedure, as pericoronitis, odontogenic cysts and tumors associated or not with the third molar, trauma in the region, or any symptoms that indicate the presence of infection.
- Presence of any systemic disorder as well diabetes, hypertension, hyperthyroidism, osteoporosis, gastrointestinal diseases that compromise the result of the surgery or any disease which impedes the use of drugs prescribed in this study.
- Patients which used any drug in the last 30 days previously to the surgical procedure.
- Patients with hypersensitivity of any drug used in this study.
- Patients who have intolerance to the other materials that will be used in the research, such as 0.5% alcoholic chlorhexidine solution, 0.12% chlorhexidine gluconate solution and 4% articaine hydrochloride solution with epinephrine 1: 100,000.
- Female patients which were in the menstrual period or pregnant period and lactation. (The menstrual period causes progesterone to act directly on the neurotransmitters GABA (gamma-aminobutyric acid), opioids, serotonin, and catecholamine, in addition to leading to an increase in prolactin, altering glucose metabolism, the function of the hypothalamic–pituitary–adrenal axis. Even causes insulin resistance and mild nutritional and electrolyte deficiencies that may lead to pain threshold, irritability, and increased fluid retention, which can bias some research analyses ^18^.

### Sample size calculation

A power test was calculated to obtain the sample size, it was based on a previous pilot study who used pain as a parameter (mean difference = 1.87; standard deviation = 1.75)^19^, which demonstrated the need for fourteen samples (teeth) for 80% of the power test, the authors added a total of sixteen samples per group. Thus, to obtain more homogeneous data (95% of the power test), 20 samples (teeth) were selected for each group, totaling 60 patients. The test was applied by SigmaPlot 12.0 (Exakt Graphs and Data Analysis, San Jose, CA, USA).

### Experimental groups

Thus, a total of one hundred teeth from 64 subjects were divided into five groups (n = 20): DEX, which represented subjects who received 8 mg of dexamethasone (Laboratório Teuto Brasileiro s/a—17,159,229,000,176, Anápolis, GO, Brazil, code: 12,804—[797728]); DEX + ETO, which represented subjects who received 8 mg of dexamethasone plus 300 mg of etodolac (Laboratório Apsen Farmacêutica, São Paulo, Brazil); DEX + LOX, which represented subjects who received 8 mg of dexamethasone plus 60 mg of loxoprofen (Daiichi Sankyo Brasil, Barueri, Brazil); DEX + KET, which represented subjects who received 8 mg of dexamethasone plus 10 mg of ketorolac (União Química, São Paulo, Brazil); and DEX + IBU, which represented subjects who received 8 mg of dexamethasone plus 600 mg of ibuprofen (Prati Donaduzzi, Toledo, Brazil). All drugs were administered one hour before surgery and administered orally. The surgical interventions were timed from the beginning of the incision until the end of the last suture.

### Randomization

Subject randomization was performed by a researcher (L.P.F.), who selected groups using the envelope system. The envelope contained papers on which the five groups’ names were written, and a random selection was performed. If the subject presented with two lower third molars, which was compatible with the inclusion criteria, the subject underwent a double draw and could be allocated to two groups. In those cases, another envelope had two papers identifying the side of the mandibular third molar: right or left, determining the sequence of the groups to be operated. The drugs were given to the subjects by the same researcher. All the drugs were manipulated, the capsules were equally the same and were given in a surgical envelope to avoid the recognize of the drug by the patient and create a bias. The surgeon (G.A.C.M.) and the researcher who conducted the analysis (T.O.B.P.) did not know the groups to groups to which the subjects were assigned, characterizing a *triple-blind* clinical trial.

When a subject had bilateral third molars, the choice to initially operate on the right or left side was also determined by the researcher (L.P.F.) using the envelope system. In this situation, the surgeries were conducted unilaterally with a minimum interval of 21 days between the extraction procedures.

### Surgical procedures

The surgical procedures were performed by the same surgeon. All subjects received preoperative antibiotic therapy with 2 g of amoxicillin 1 h before the surgical procedure. Intrabuccal antisepsis was performed with vigorous mouth washing for 1 min with an aqueous solution of 0.12% chlorhexidine digluconate, followed by extraoral antisepsis with 0.5% chlorhexidine alcohol solution. The anesthetic technique employed comprised a regional blockade of the inferior alveolar, buccal, and lingual nerves. This was performed using a reflux syringe with a long 27-gauge gingival needle for injection of 2% mepivacaine hydrochloride with epinephrine 1:100,000 (Mepiadre, DFL) at a maximum volume of 4.5 ml, equivalent to 2.5 tubes.

A triangular linear flap was made with a number 15 scalpel blade in the distal region of the lower second molar in association with a buccal releasing incision in the mesial aspect of the second molar. Following this, mucoperiosteal detachment was performed with periosteal relief and a retractor was used to expose the operative field. Osteotomy was performed using a model 702 carbide-tipped drill bit mounted on a high-speed pen, in addition to abundant irrigation with sterile saline NaCl 0.9%. Dental removal was completed with curved and straight extractors of the Seldin type (n° 2, 1R, or 1L), followed by careful inspection for the removal of pericoronal follicles with curettes and Kelly curve tweezers.

The bone margins were trimmed to remove bone spicules using a bone file, with abundant irrigation using sterile saline NaCl 0.9%. Thereafter, sutures were performed with nylon 5.0 (Ethicon, Johnson & Johnson, Brazil). Surgical interventions were performed in the morning and afternoon (between 08 am and 6 pm) in a temperature environment control.

After the surgical procedure, all subjects received 12 tablets of 750 mg paracetamol (not identified to the subject, in white tablet form) and were advised to take 1 tablet in case of pain, with a minimum interval of 6 h between administrations of the analgesic. Subjects were instructed to recording a log of all relevant variables such as VAS score at each timepoint, and when analgesics were taken.

Immediately after the surgical procedures, the subjects were prescribed a cold, liquid, pasty, hyperprotein-based diet for the first 48 h postoperatively, as well as other aspects of general care, such as avoiding physical exertion and sun exposure. Subjects were advised not to use cold compresses during the postoperative period. Any subject who developed postoperative complications, such as bleeding and alveolar osteitis was treated and consequently excluded from the study. Follow-up visits were scheduled at 48 h and 7 days postoperatively to evaluate for edema and MMO. Sutures were removed at the 7-day postoperative mark.

### Primary outcome

For this study, the primary outcome variable was the assessment of pain (visual analog scale VAS). Number of rescue analgesics (NAR), Salivary PGE2 concentration, edema and MMO were secondary outcomes variables.

Pain was measured using a visual analog scale (VAS). During the postoperative period, all subjects received a VAS protocol containing a linear chart with a scale from 0 to 10. The subjects were instructed to point out the level of postoperative pain on the chart at 6, 12, 24, 48, and 72 h and 7 days after the surgical procedure. A VAS score of 0 represented the absence of postoperative pain, whereas a score of 10 represented the worst pain the subject had experienced^12,20^. The weighted average corresponding to the values of every subject in each group was obtained.

Another outcome measure for pain involved the number of analgesics consumed by the subject during postoperative period. Subjects were instructed to record the time of first analgesic consumption. The patients were no induced to consumed or not the drugs, only were informed that were usually drugs destined to relieve pain, edema and trismus and might be used only if were extremely necessary for a rescue way. The weighted average corresponding to the values of every subject in each group was obtained^21^.

The salivary collection was performed before subjects took the preoperative drug, prior to the surgical procedure, and 48 h after the surgical procedure. Saliva was collected directly in a salivate tube (Salivette; Genese Produtos Diagnósticos Ltda, São Paulo, SP, Brazil). The subjects were informed not to eat or brush their teeth in the 2 h preceding the collection procedure. The tubes were properly capped and stored at −20 °C to keep the samples stable. The salivates were centrifuged at 1000 g for one minute, resulting in a clean and fluid saliva sample that was used to determine the PGE2 salivary concentration. Salivary PGE2 was measured by competitive enzyme-linked immunosorbent assay (ELISA) method using a commercial kit (Diametra DKO020, Milano, Italy).

**Figure 1:**
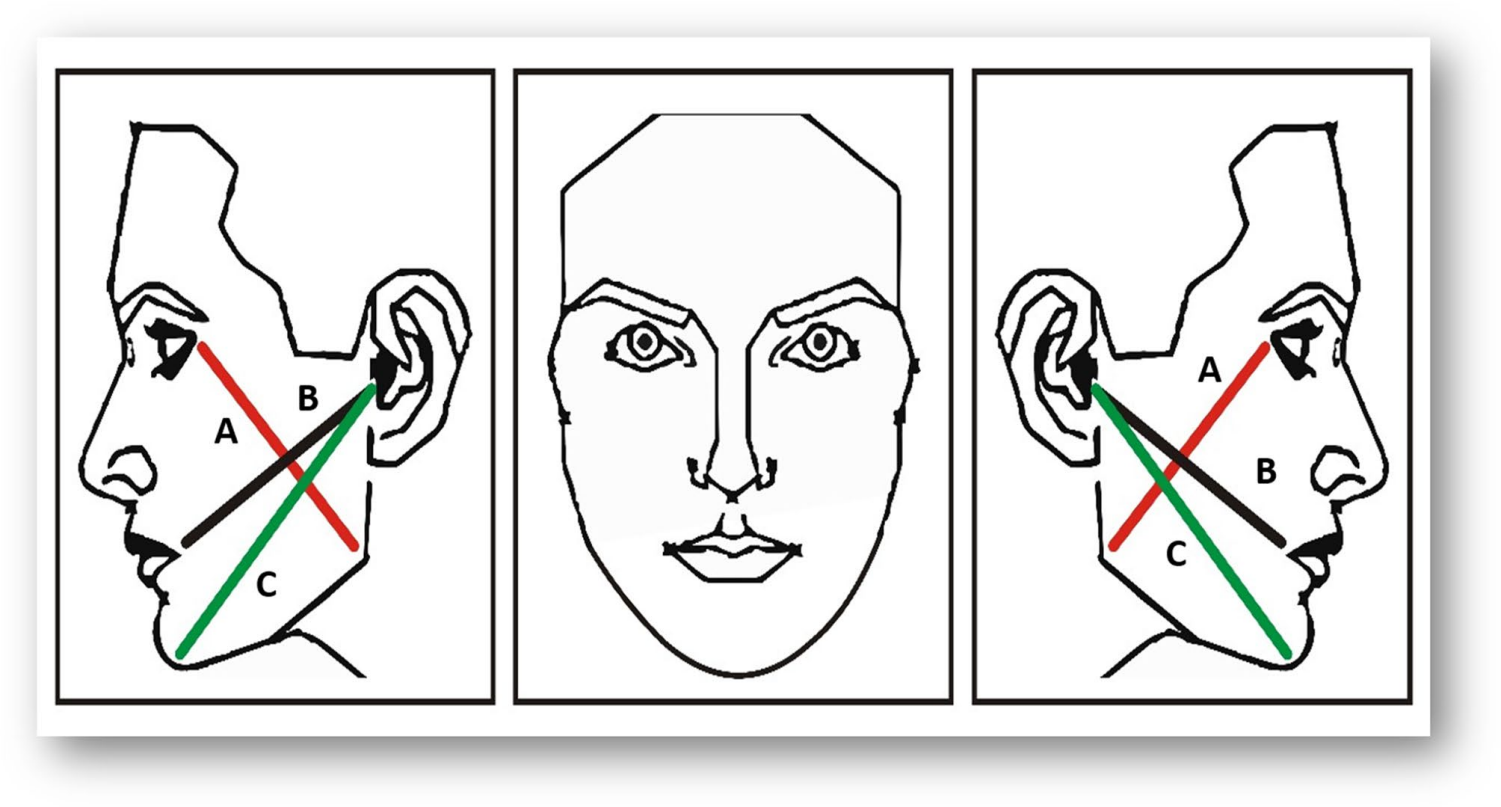
Representative scheme showing the topographic points measured for edema evaluation. The line A represented the point from mandibular angle up to corner of the eye. Line B represented the point from tragus up to labial commissure and line C represented the point from tragus up to pogonion. (CorelDRAW Graphics Suite X7.6- https://www.coreldraw.com).

Edema measurement was performed using a three-point analysis with a tape measure. Measurements were made of the mandibular angle to the lateral canthus, tragus to labial commissure, and tragus to pogonion at the preoperative, 48 h postoperative, and 7 days postoperative periods (Fig. 1)^22^.The values obtained were recorded in the subjects’ individual files. The researcher (T.O.B.P.) who performed these measurements was blinded and also determined all the results of the analyses of this study. To test intra-observer variability and increase measurement precision, all measurements were repeated by the same operator 3 times to ensure reliability of the method and consistency of the operator^23^. To assess postoperative edema, an average of the three-point measurements was calculated at 48-h postoperative and 7-day postoperative.

Maximum mouth opening was evaluated using measurements in millimeters made with a digital caliper (Mitutoyo, Sakado, Japan) between the incisal edge of the upper central incisors and the lower central incisors on the right side. The measurements were performed preoperatively, as well as 48 h and 7 days after the surgical procedure^12,20^.

For the calibration of the researcher, four patients were selected to MMO and edema measurements, before the beginning of the study. The analysis was performed by the same researcher twice in a 21 days of interval. The Kappa test showed the concordance intra-examiner (K > 0.95).

### Follow-up

The follow-up time of the patients after the analyses (7 days) varied from 2 to 3 months after surgery.

### Statistical analysis

All quantitative data underwent a statistical analysis and normality statistical testing (Shapiro–Wilk test) for homogeneity or heterogeneity distribution. For VAS, edema, and MMO, a two-way ANOVA and Tukey posttests were applied, and results were considered significant when *P* < 0.05. For PEG2 concentration, a one-way ANOVA and Tukey posttests were performed, results were considered significant when *P* < 0.05. Only NRA failed in the homoscedasticity statistical test, and a Kruskal–Wallis test was applied. An intention to treat analysis was performed for all randomized patients who were included in this study.

### Ethical approval

All procedures performed in studies involving human participants were in accordance with the ethical standards of the institutional and/or national research committee (#818.680) and with the 1964 Helsinki declaration and its later amendments or comparable ethical standards.

### Informed consent

Informed consent was obtained from all individual participants included in the study.

## Results

### Demographic data

**Figure 2:**
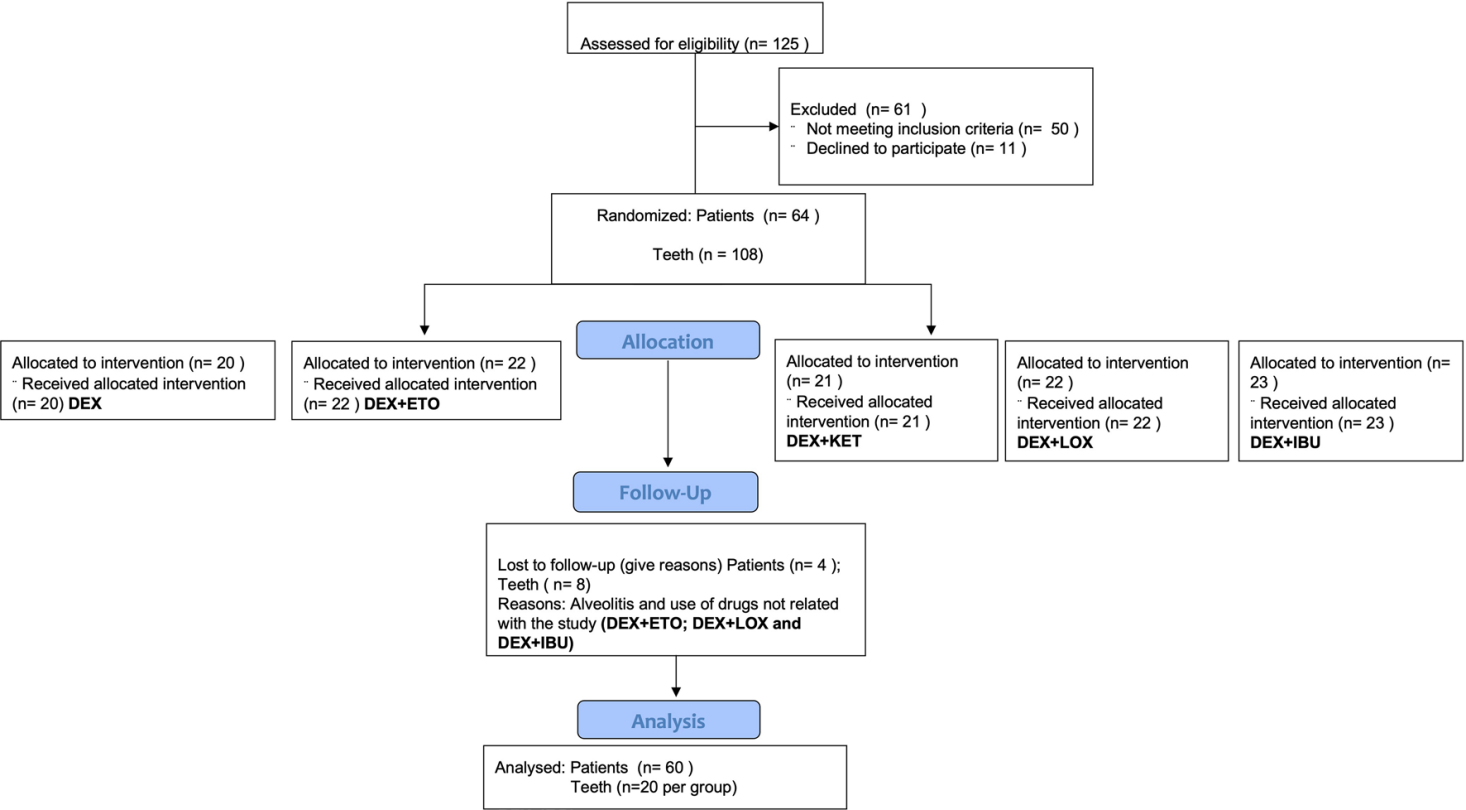
Flowchart of subjects screened according to CONSORT statement.

A total of 125 subjects were screened for eligibility. Sixty-one subjects were excluded. Fifty subjects did not meet the inclusion criteria and 11 subjects declined to participate in the study. Thus, 64 subjects were randomized and divided into five groups. All 64 subjects received the planned intervention, however, at the follow-up visit, four subjects were excluded due to postoperative dry socket alveolitis and the use of drugs/medicaments not related to the study as described in the exclusion criteria (Fig. 2). Demographic data regarding mean age, sex, and number of teeth removed are shown in Table 1.

**Table 1:** Characteristics of the patients.

Variable	Value
Number of patients	60
**Age (years)**	
Mean ± SD	25.36 ± 48.74
Range	18–35
**Sex (%)**	
Male	18%
Female	82%
**Teeth (patients)**	
38	24
48	40
38 and 48	36
Total	100

### Visual analog scale (VAS)

For the multiple comparison in VAS data, the drugs and periods showed a statistically significant difference (*P* < 0.001, ANOVA test). During the first six hours, DEX + ETO (mean ± standard deviation—SD) (1.9 ± 0.3) and DEX + KET (2.1 ± 0.4) decreased pain perception significantly, compared with DEX alone (5 ± 0.6) *P* = 0.006 and *P* = 0.032, respectively. The comparison between DEX + IBU (4.9 ± 0.34) and DEX + LOX (3.9 ± 0.42) with DEX showed similar pain perception (*P* < 0.05; Tukey test).

At 12 h postoperative, DEX + ETO (2 ± 0.35) and DEX + KET (2.1 ± 0.45) continued to decrease pain perception, but at this point, DEX alone (2.9 ± 0.64) showed similar values (P > 0.05; Tukey test). DEX + IBU (3.9 ± 0.87) and DEX + LOX (3.8 ± 0.76) still showed high pain perception values at this time.

**Figure 3:**
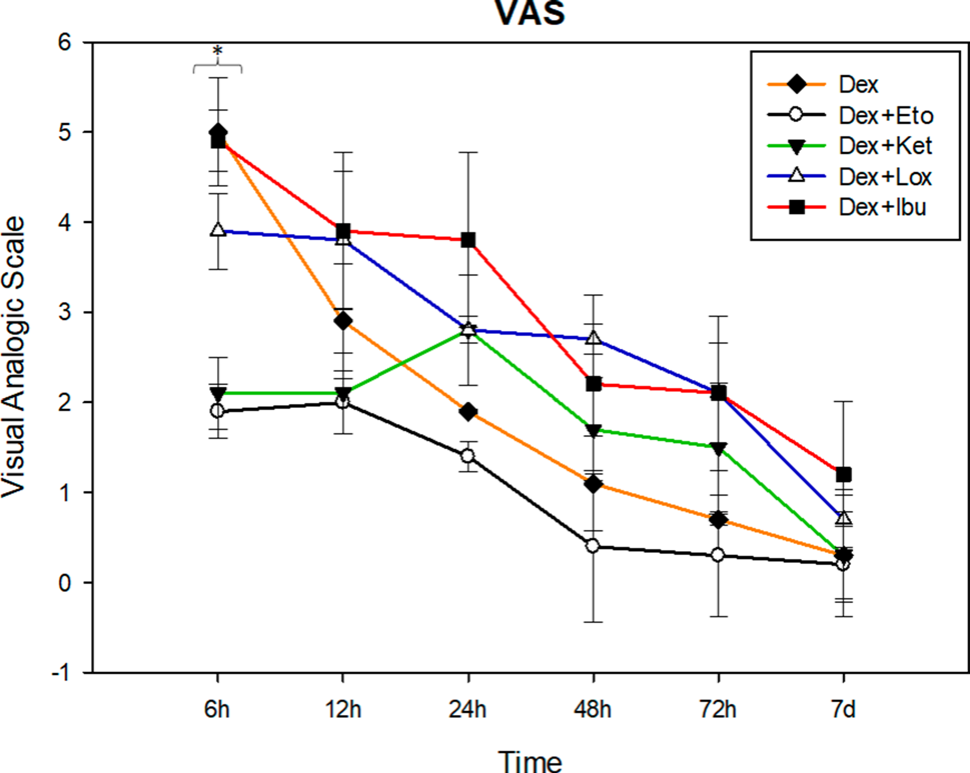
Data regarding VAS analysis. *It was possible noticed that at the first 6 h postoperative, pain perception from DEX + ETO and DEX + KET groups were significantly lower than DEX and DEX + IBU (*P* < 0.05—Tukey test). The other postoperative periods showed no significant difference between groups.

After the first postoperative day (24 h, 48 h, 72 h, and 7 days), DEX + ETO showed the lowest pain perception values. However, DEX showed similar values (*P* > 0.05; Tukey test). DEX + KET maintained its previous average, and DEX + IBU and DEX + LOX showed the worst pain perception values during all postoperative periods (Fig. 3).

### Number of rescue analgesics (NRA)

**Figure 4:**
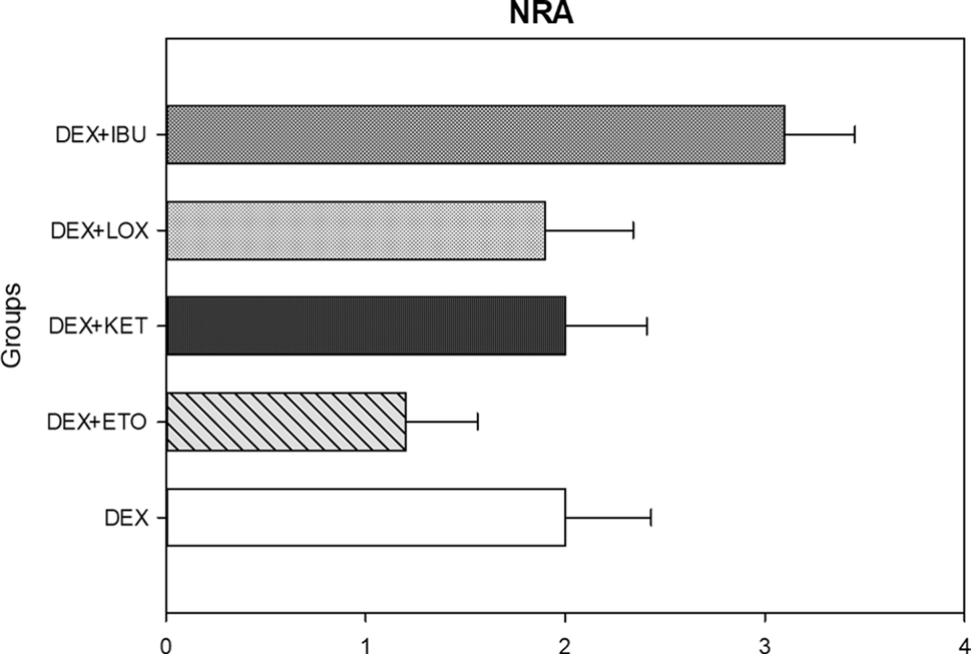
Chart regarding number of rescue analgesic consumption for 7 days postoperative. No significant difference was found for any comparison among groups (*P* = 0.267; ANOVA test).

NRA data showed in DEX + ETO group an average consumption of 1.2 analgesics during the entire postoperative recovery period (1.2 ± 0.36). After this group, subjects in the DEX (2 ± 0.43) and DEX + KET (2 ± 0.41) groups an average of 2 analgesics during postoperative recovery period. Subjects in the DEX + LOX group consumed an average of 1.9 analgesics (1.9 ± 0.44). Subjects in the DEX + IBU group consumed an average of 3 analgesics (3 ± 0.35) during the postoperative recovery period (Fig. 4). No significant difference was found for any comparison among groups (*P* = 0.267; ANOVA test).

### Prostaglandin E2 salivary concentration (PGE2)

**Figure 5:**
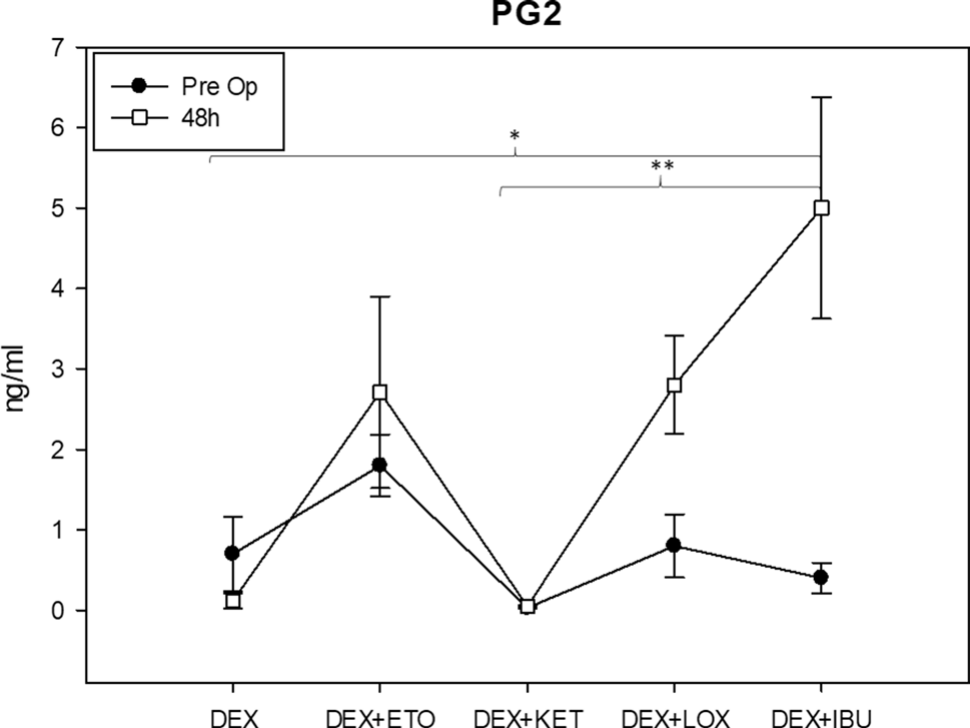
Chart regarding prostaglandin salivary E2 (PGE2) concentration. Data showed that DEX group was the only which decreased the PGE2 at 48 h postoperative (80%), while other groups showed an increase of PGE2 concentration at 48 h postoperative. *The value indicated significant differences between the DEX and DEX + IBU groups (*P* < 0.05—Tukey test). ** The value indicated significant differences between the DEX + KET and DEX + IBU groups (*P* < 0.05—Tukey test).

Data about the salivary concentration of prostaglandin E2 (PGE2) 48 h after the surgical procedure indicated that the DEX group (average 0.2 ng/ml) was the only one in which there was a decrease of concentration of PGE2 compared to the preoperative period (average 0.7 ng/ml). Subjects who only took DEX one hour before the surgery showed an 80% decrease in PGE2 48 h after surgery. All other groups showed an increase in PGE2 concentration 48 h after surgery. Subjects who took DEX + KET showed a 66.6% increase in PGE2 concentration. This data was followed by PGE2 concentration levels for the DEX + ETO (99.2% increase; Preoperative: 1.8 ng/ml; postoperative: 2.7 ng/ml), DEX + LOX (339.2% increase; Preoperative: 0.8 ng/ml; Postoperative: 2.8 ng/ml) and DEX + IBU (2956.25% increase; Preoperative: 0.4 ng/ml; Postoperative: 5 ng/ml) groups. Values for 48-h postoperative salivary PGE2 concentration indicated significant differences between the DEX and DEX + IBU groups (*P* = 0.003—Tukey test) and between DEX + KET and DEX + IBU (*P* < 0.001—Tukey test) (Fig. 5).

### Edema

**Figure 6:**
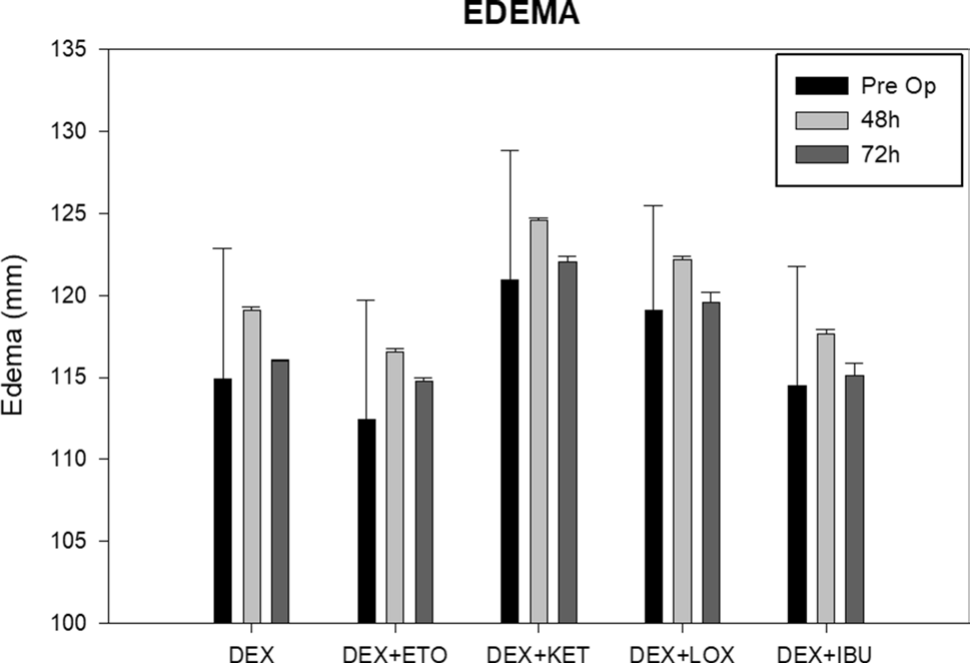
Chart regarding postoperative edema (mm). There was no statistical difference among the groups (*P* > 0.05—Tukey test).

The DEX + LOX (3.1 ± 0.2 mm) and DEX + IBU (3.12 ± 0.32 mm) groups showed lower numerical values for edema 48 h after the surgical procedure. These groups were followed by DEX + KET (3.6 ± 0.2 mm) and DEX + ETO (4.1 ± 0.2 mm), which showed similar values to DEX alone (4.2 ± 0.2 mm). After 7 days, all groups showed low values for edema except DEX + ETO (2.2 ± 0.2 mm). There was no statistical difference among groups (*P* > 0.05—Tukey test) (Fig. 6). Only the source time of analysis showed significant changes, in which the edema increased in the first 48 h postoperative, and decreased, at 7 days postoperative, similar to preoperative data.

### Maximum mouth opening (MMO)

**Figure 7:**
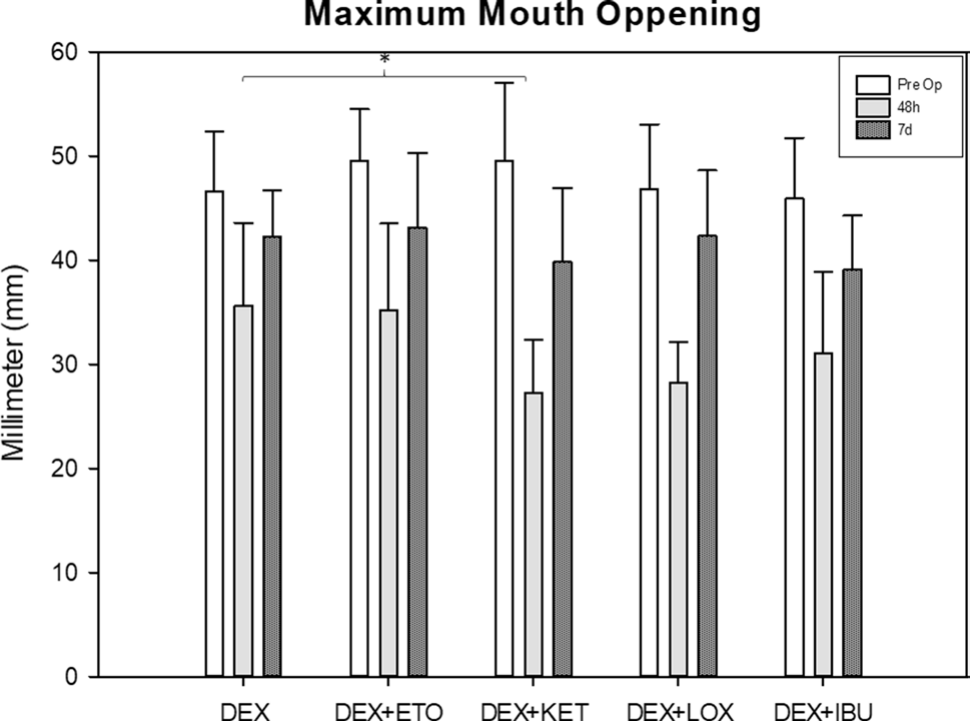
Chart regarding maximum mouth opening (mm). It was observed that DEX + KET group showed significant lower MMO at 48 h postoperative compared to DEX group (*P* < 0.05—Tukey test). Other groups showed no significant difference on postoperative MMO.

Only for the source of variation "time of analysis" there was a significant difference (*P* < *0.001*—ANOVA test). The DEX + KET group (27.2 ± 5.18 mm) showed the lowest values for 48 h postoperative MMO (*P* < *0.05*—Tukey Test). The DEX group (35.6 ± 7.96 mm) showed higher values for 48 h postoperative MMO (*P* < 0.05 – Tukey test). The other groups showed similar values compared to the DEX group (*P* > 0.05; DEX + ETO: 35.2 ± 8.33; DEX + IBU: 31 ± 7.83; DEX + LOX: 28.2 ± 3.88). After 7 days, all groups showed a decrease in trismus (or increase in MMO), showing similar values for all comparisons (*P* > 0.05) (Fig. 7).

## Discussion

This study aimed to evaluate whether benefits exist with a combination of NSAIDs plus DEX in decreasing postoperative pain, edema, and MMO following third molar surgery. Prior studies are unclear whether the combination of these drugs could provide an advantage in postoperative period, which justifies the clinical use of various drugs preoperatively. However, the combination of some NSAIDS plus DEX seems to improve symptoms in the first postoperative hours of acute pain.

The preemptive use of steroids for third molar surgery, mainly DEX, is well established, and provides positive results regarding a comfortable postoperative period for the patients^8,24,25^. A 2008 clinical study by Laureano-Filho and colleagues^19^ compared a preoperative dose of 4 mg and 8 mg of DEX in third molar surgeries and observed that there was a significant difference between the doses regarding postoperative MMO and edema, but not postoperative pain, as was observed in another study^26^. This lack of effect on pain may be explained by the ineffectiveness of DEX in reducing prostaglandins at the site of an injury^27^. Another possible explanation for this phenomenon is that cortisol naturally inhibits pituitary beta-endorphins, which are potent endogenous analgesic present in the peripheral circulation^28^.

Some clinical trials have suggested that the combination of steroids and NSAIDS improves preemptive effects on MMO, edema, and pain compared to steroids alone^4,14^. This study found that the combination of DEX plus several NSAIDS (DEX + ETO, DEX + KET, DEX + IBU, and DEX + LOX) showed no significant difference in the edema and MMO parameters. The exception was the combination of DEX + KET, which showed a significantly lower value for 48-h postoperative MMO compared with DEX alone. These results could be explained because, as noted above, the use of preoperative DEX alone is well established and provides positive results regarding the improvement of postoperative edema and MMO^8,24,25^.

Although data were obtained about postoperative pain, the VAS analysis showed that patients who took preoperative DEX + ETO and DEX + KET exhibited significantly lower pain perception during the first 6 postoperative hours compared with all the groups. At 12 h postoperative, the DEX group showed similar values to the DEX + ETO and DEX + KET groups, whereas the DEX + LOX and DEX + IBU groups maintained higher pain values during all periods. After the first day (24 h), subjects who took DEX + KET showed an increase in pain perception compared with the DEX and DEX + ETO groups, which had lower VAS values during the postoperative period. There was no significant difference between these two groups.

NRA did not significantly differ among the groups; however, it seemed to be lower for the DEX + ETO (average of 1.2 postoperative analgesics) group compared to the other groups. In addition, the DEX + IBU group presented the highest consumption of all groups. However, it is important to highlight that even though NRA was higher for DEX + IBU group, subjects who received this drug preoperatively took an average of three analgesics during the seven postoperative days. This value is well below what is usually prescribed in most practices, which also often include three days of acetaminophen with opioids at an interval of 6 h^29,30^.

DEX alone was the only preoperative therapy that decreased the salivary expression of PGE2 48 h after the surgical procedure. The other groups showed an increase in salivary PGE2. However, the DEX + ETO and DEX + KET groups showed a discreet increase, whereas the DEX + IBU and DEX + LOX groups showed a significant increase in PGE2. This data disagrees with the VAS, which showed that the DEX, DEX + ETO, and DEX + KET groups presented similar pain perception values during the entire postoperative period. However, PGE2 is not the only pain mediator released during an inflammation response. Other algogenic substances are generated from arachidonic acids, such as bradykinin, leukotrienes, tumoral necrosis factors (TNF-α) and interleukins^31^. Although the PGE2 expression values were higher, VAS evaluated the subjects’ pain perception. Thus, even though the drugs had no effect on PGE2 expression, they might have decreased the subjects’ pain perception. Laureano-Filho and colleagues^19^ showed that 8 mg of DEX 1 h before surgery decreased the expression of PGE2 and thromboxane A2 at the surgical site, but this had a minimal effect on reported pain the day of the surgery. Similar results were found in another trial with methylprednisolone 125 mg^1^.

There is no consensus about the preemptive combination of DEX and an NSAID. Although trials have shown great results for this therapy, this study observed that not all combinations produced good results. DEX + IBU and DEX + LOX presented poor results for all parameters, and DEX alone seems sufficient to relieve postoperative pain. Currently, the medical field seeks to decrease the number of drugs prescribed to patients. Furthermore, NSAIDs have several adverse effects, such as cardiovascular and gastrointestinal events^32^. Preemptive DEX alone performed well for postoperative pain, edema, MMO and, decreased salivary PGE2, but KET and ETO, when combined with DEX in a preemptive way, showed similar results to DEX alone in terms of VAS scores 7 days after the surgical procedure and significantly decreased patients’ pain perception at 6 h after surgery, compared to DEX alone, which resulted in the worst acute pain after the procedure. This demonstrates important benefits for patients’ postoperative comfort.

Indeed, ETO and KET have proven effective against pain when prescribed after third molar surgeries^12,33,34^. When combined with DEX, a single preoperative dose of KET and/or ETO seems to significantly reduce acute pain during the first few hours, and there is no need to prescribe any postoperative NSAID except a rescue analgesic. This study presented some limitations. It did not account for other allogenic cytokine expressions did not evaluate other NSAIDs, so more trials should be designed to establish a clinical consensus about this field. However, the use of specific NSAIDs combined with 8 mg of DEX after third molar surgeries might provide patients with postoperative benefits and should be considered in the dentistry routine.

## Conclusion

It can conclude that the preemptive use of steroids with NSAIDS assessed in this study, the combinations involving KET and ETO, significantly improved patients’ postoperative pain during the first few hours.
